# Total Neoadjuvant Therapy Versus Standard Neoadjuvant Chemoradiotherapy for Borderline Resectable Pancreatic Cancer: A Systematic Review and Meta-Analysis

**DOI:** 10.7759/cureus.115551

**Published:** 2026-08-31

**Authors:** Sonam Gupta, Satya Sarangi, Ashutosh Jaiswal, Mohd Riyaz Lattoo, Sajjan Rajpurohit, Deepak Mittal, J. C. Alepes Lamin, Tarun Durga, Rajan Yadav, Younis Ahmad

**Affiliations:** 1 Department of Gastrosurgery, Medanta Hospital, Noida, IND; 2 Department of Medical Oncology, Medanta Hospital, Noida, IND; 3 Department of Gastrosurgery, Sher-i-Kashmir Institute of Medical Sciences (SKIMS), Srinagar, IND; 4 Department of Radiation Oncology, Medanta Hospital, Noida, IND; 5 Department of Radiation Oncology, Civil Hospital, Shillong, IND; 6 Department of Medical Oncology, Medanta Noida, Noida, IND; 7 Department of Medical Oncology, Gujarat Cancer and Research Institute (GCRI), Ahmedabad, IND; 8 Department of Gastrosurgery, Government Medical College (GMC), Srinagar, IND

**Keywords:** meta-analysis, pathological complete response, r0 resection rate, resectable pancreatic cancer, total neoadjuvant therapy

## Abstract

The optimal neoadjuvant strategy for borderline resectable pancreatic cancer (BRPC) remains uncertain. Total neoadjuvant therapy (TNT) combines extended induction chemotherapy with radiotherapy before surgery. Whether TNT improves oncologic outcomes over standard neoadjuvant chemoradiotherapy (NACRT) is unclear; the incremental benefit of the radiotherapy component itself has also been questioned. A systematic search of PubMed, Embase, Cochrane CENTRAL, Web of Science and Scopus identified comparative studies of TNT vs standard NACRT in BRPC published between January 2005 and May 2026. Randomized controlled trials and observational comparative studies were included. Random-effects meta-analysis using the DerSimonian-Laird method was performed. Primary outcomes were overall survival and R0 resection rate. Secondary objectives assessed the independent survival benefit of radiotherapy and the influence of chemotherapy backbone selection. Twenty-three studies met inclusion criteria for qualitative synthesis. Nineteen primary studies comprising 9,219 patients contributed to data extraction. TNT was associated with superior R0 resection rates (94%-95% vs. 78%-80%) and higher pathological complete response (pCR) rates (5.8%-13.0% vs. 2.1%-6.0%) compared to standard NACRT. TNT was associated with improved overall survival in the largest available database analysis (hazard ratio (HR), 0.82; 95% confidence interval (CI), 0.72-0.94), though a smaller cohort showed no difference. The radiotherapy component did not confer an independent survival benefit when added to systemic chemotherapy (PREOPANC-2 BRPC subgroup HR 0.80, 95% CI 0.53-1.21, *P *= 0.30). PAXG demonstrated superior event-free survival over mFOLFIRINOX (HR, 0.63; *P *= 0.0018). Grade 3 to 4 adverse events were frequent (60%-67%), but surgical morbidity was comparable across treatment arms. Findings suggest that TNT improves pathologic response and margin-negative resection rates compared to standard NACRT in BRPC. There appears to be modest improvement in survival but is not uniformly reproduced. The survival advantage appears driven primarily by extended systemic chemotherapy rather than the radiotherapy component. The choice of chemotherapy backbone significantly influences event-free survival.

## Introduction and background

Pancreatic ductal adenocarcinoma (PDAC) carries a five-year overall survival (OS) rate of ~12%. Borderline resectable pancreatic cancer (BRPC) occupies a clinically critical position between clearly resectable and locally advanced disease. Tumor abutment or encasement of major mesenteric vasculature renders curative resection technically feasible but at high risk of positive margins. Neoadjuvant therapy has become the standard of care for BRPC. Versteijne et al. established that gemcitabine-based chemoradiotherapy (CRT) improves long-term OS vs. upfront surgery, with a five-year OS of 20.5% vs. 6.5% (hazard ratio (HR), 0.73; 95% confidence interval (CI), 0.56-0.96) [1]. This finding confirmed that preoperative treatment improves outcomes in BRPC. The question has since shifted from whether to treat before surgery to how best to do so.

Two broad neoadjuvant strategies have emerged. Standard neoadjuvant CRT (NACRT) delivers a short course of concurrent chemoradiation, typically with gemcitabine or a fluoropyrimidine, and is followed by surgery and adjuvant chemotherapy [2,3]. Total neoadjuvant therapy (TNT) sequences extended multi-agent induction chemotherapy before radiotherapy and surgery, with no planned adjuvant treatment [2,3]. The rationale for TNT rests on earlier control of occult systemic disease, prolonged treatment observation that filters out patients with unfavorable biology before irreversible surgery, higher rates of pathologic response, and guaranteed delivery of all intended systemic therapy.

The PREOPANC-2 trial compared neoadjuvant FOLFIRINOX against gemcitabine-based CRT in resectable and borderline resectable PDAC [4,5]. No significant difference in OS was observed (21.9 vs. 21.3 months). This result raised a fundamental question: if extended chemotherapy alone produces survival equivalent to CRT, does the radiation component of TNT add meaningful benefit? Further, the ESPAC-5F trial compared four neoadjuvant strategies, including both chemotherapy-alone and CRT arms in BRPC [6]. The CASSANDRA trial reported that the four-drug PAXG regimen was superior to mFOLFIRINOX in event-free survival [7]. Wu et al. conducted a comprehensive meta-analysis of 31 prospective studies and found that neoadjuvant chemotherapy alone was associated with significantly longer OS than chemotherapy combined with CRT (25.6 vs. 17.6 months) [8]. The survival disadvantage of CRT disappeared when five or more cycles of induction chemotherapy preceded radiation. This conditional relationship between induction chemotherapy duration and radiation benefit has not been incorporated into a BRPC-specific meta-analysis.

Rationale, knowledge gap, and objective

No systematic review has synthesized the comparative evidence on TNT vs. standard NACRT specifically in BRPC, nor addressed the independent contribution of radiotherapy to survival in this population. This meta-analysis aims to fill that gap. We present this article in accordance with the Preferred Reporting Items for Systematic Reviews and Meta-Analyses (PRISMA) reporting checklist.

## Review

Methodology

Adherence to PRISMA Guidelines

This systematic review was conducted in accordance with the PRISMA 2020 guidelines [9].

Eligibility Criteria

Studies were eligible if they compared TNT with standard NACRT or compared neoadjuvant chemotherapy combined with radiotherapy vs. chemotherapy alone in adult patients with borderline resectable PDAC. Eligible study designs included randomized controlled trials (RCTs), quasi-randomized trials, prospective cohort studies, and retrospective comparative studies. Single-arm studies, case reports, case series, editorials, narrative reviews, and conference abstracts with insufficient data were excluded. Studies combining BRPC with locally advanced or resectable PDAC were included only when BRPC subgroup data were separately reported or extractable. Borderline resectability was defined according to National Comprehensive Cancer Network (NCCN) [10], American Hepato-Pancreato-Biliary Association (AHPBA)/Society for Surgery of the Alimentary Tract (SSAT)/Society of Surgical Oncology (SSO) [11], or MD Anderson consensus criteria [12]. The search was restricted to English-language publications from January 2005 to May 2026.

Information Sources and Search Strategy

Systematic searches were performed in PubMed/MEDLINE, Embase (via Ovid), Cochrane CENTRAL, Web of Science Core Collection, and Scopus. The search strategy combined terms for pancreatic cancer, borderline resectable, neoadjuvant therapy, and CRT using both controlled vocabulary and free-text terms. The strategy was peer-reviewed using the Peer Review of Electronic Search Strategies (PRESS) checklist. Full search strategies are provided in the supplementary material. Additional sources included ClinicalTrials.gov and World Health Organization-International Clinical Trials Registry Platform (WHO-ICTRP) for registered trials; reference lists of included studies and relevant systematic reviews; forward citation tracking using Google Scholar; and conference proceedings from American Society of Clinical Oncology (ASCO), ASCO Gastrointestinal Cancers Symposium (ASCO GI), European Society for Medical Oncology (ESMO), Americas Hepato-Pancreato-Biliary Association (AHPBA), and International Hepato-Pancreato-Biliary Association (IHPBA) (2015-2026) [13-19].

Study Selection

All records were imported into Covidence, and duplicates were removed. Two reviewers independently screened titles and abstracts against the eligibility criteria. Full-text review was performed independently by both reviewers. Disagreements were resolved by discussion or by a third reviewer.

Data Extraction

Two reviewers independently extracted data using a standardized, pilot-tested form. Study-level characteristics, patient demographics, intervention details, and outcome data were extracted. For time-to-event outcomes, HRs with 95% CIs were extracted directly where reported. Where HRs were unavailable, they were estimated from Kaplan-Meier curves using the Tierney method. Dichotomous outcomes were extracted as event counts with denominators.

Outcomes

Primary outcomes were OS and R0 resection rate. Secondary outcomes included progression-free survival or disease-free survival, overall resection rate, pathologic complete response (pCR) rate, treatment completion rate, grade 3-4 adverse events (AEs), major postoperative morbidity (Clavien-Dindo grade III or higher [20,21]), 90-day mortality, locoregional recurrence, and distant metastasis. Two secondary objectives were prespecified: (1) whether the addition of radiotherapy to systemic chemotherapy independently improves survival in BRPC; and (2) whether the choice of chemotherapy backbone influences outcomes.

Risk-of-Bias Assessment

Risk of bias (RoB) was assessed independently by two reviewers. The Cochrane RoB 2 tool was applied to RCTs across five domains [22]. The Newcastle-Ottawa Scale (NOS) was used for observational studies [23].

Statistical Analysis

Random-effects meta-analysis using the DerSimonian-Laird method was performed where two or more studies reported the same outcome with sufficient clinical homogeneity. HRs were used for time-to-event outcomes. Odds ratios were calculated for dichotomous outcomes using event counts. Heterogeneity was assessed using Cochran's Q test (significance threshold *P *< 0.10) and the I-squared statistic. *I*^2 ^values of 0%-25% were considered low, 26%-50% moderate, 51%-75% substantial, and above 75% considerable. Prespecified subgroup analyses included: study design (RCTs vs. observational), radiotherapy inclusion (chemo plus RT vs. chemo alone), chemotherapy backbone (FOLFIRINOX vs. gemcitabine-based), radiotherapy modality (conventional CRT vs. SBRT), geographic region, and number of induction chemotherapy cycles (five or more vs. fewer than five before RT). Publication bias was assessed using funnel plots; formal Egger's test was planned if 10 or more studies contributed to a given analysis. Statistical analyses related to the meta-analysis were performed using R version 4.4.3 (R Foundation for Statistical Computing, Vienna, Austria).

Certainty of Evidence

The certainty of evidence for each primary and key secondary outcome was assessed using the GRADE framework, rating evidence as high, moderate, low, or very low based on RoB, inconsistency, indirectness, imprecision, and publication bias.

Results

Study Selection

The database search identified 487 records and 36 additional records from other sources. After removing 134 duplicates, 389 records were screened by title and abstract. Full-text assessment was performed on 83 reports. Sixty were excluded for the following reasons: no BRPC subgroup data (*n* = 18), no comparator arm (*n* = 12), duplicate cohort (*n* = 8), conference abstract with insufficient data only (*n* = 7), insufficient outcome data (*n* = 6), not in English (*n* = 5), and review or editorial (*n* = 4). Twenty-three studies met all inclusion criteria for qualitative synthesis; 19 contributed quantitative data to the meta-analysis. The study selection process is shown in Figure 1 as a PRISMA flow diagram.

**Figure 1:**
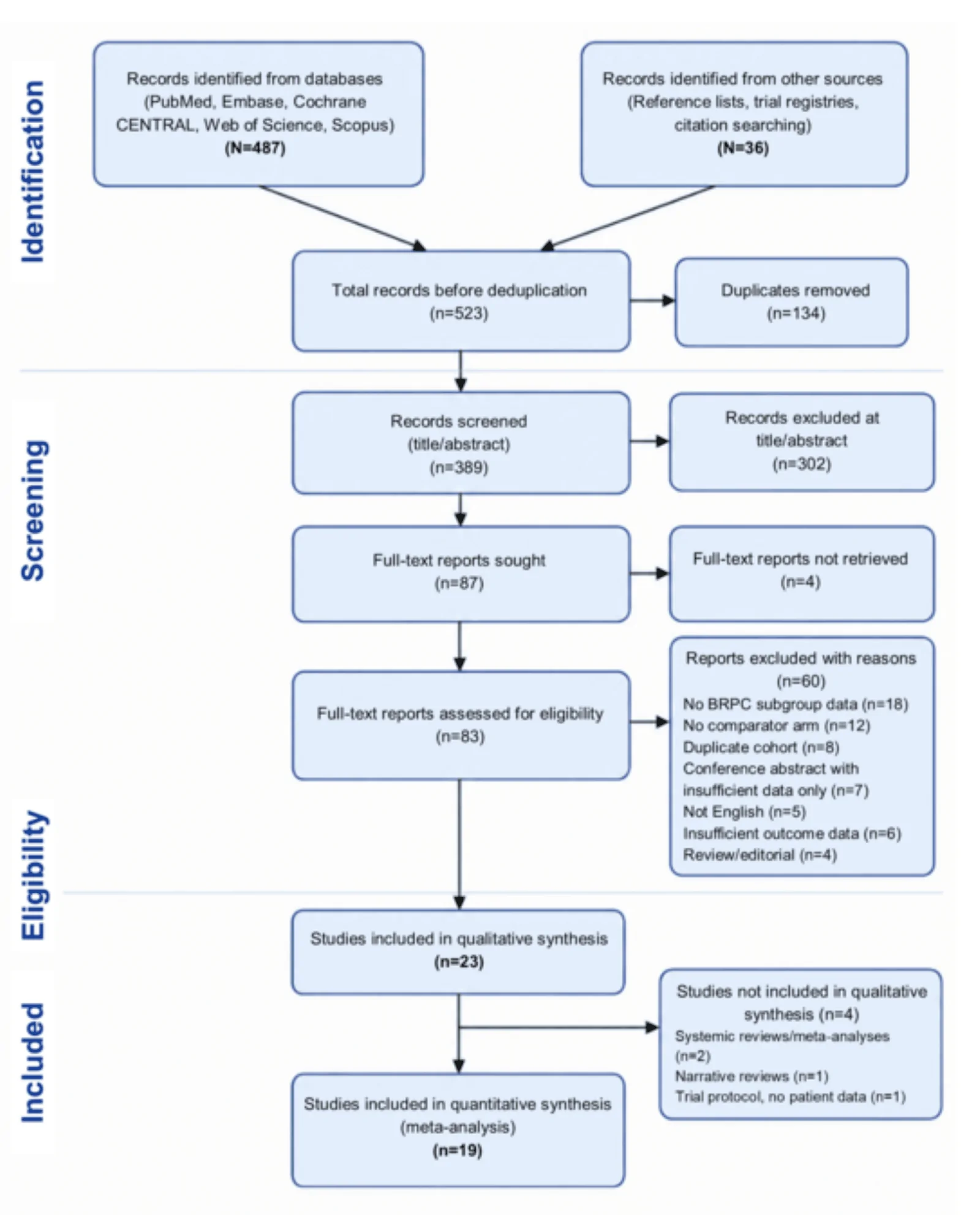
PRISMA 2020 flow diagram. BRPC, borderline resectable pancreatic cancer; PRISMA, Preferred Reporting Items for Systematic Reviews and Meta-Analyses

Study Characteristics

Twenty-three studies were included in the qualitative synthesis. Of these, 19 were primary studies with original patient data, two were secondary studies (systematic reviews/meta-analyses [8,24]), one was a narrative review [25], and one was a trial protocol without patient outcome data [26]. The 19 primary studies enrolled a combined 9,219 patients (Table 1). Cohorts originated from the United States (*n* = 7), Japan (*n* = 3), the Netherlands (*n* = 2), Australia (*n* = 2), and one each from Italy, China, India, France, and a multinational European collaboration. Publication dates ranged from 2015 to 2026. Four studies were prospective RCTs [4,6,7,27]. One additional study was a dedicated surgical sub-analysis of PREOPANC-2 [28]. The remaining 14 studies (74%) were observational. Median follow-up was reported in only five studies (range 14-42.3 months). Patient populations varied in resectability classification. Five studies exclusively evaluated BRPC [6,29-32]. The remaining studies enrolled mixed populations. Most studies applied the NCCN criteria for borderline resectability. The PREOPANC-2 trial used criteria from the Dutch Pancreatic Cancer Group [4]. The CASSANDRA trial incorporated biological borderline criteria using a CA19-9 threshold of 500 U/mL or higher [7].

**Table 1:** Study characteristics. ^†^Dekker et al. [28] is a sub-analysis of the PREOPANC-2 trial [4] using the same patient cohort. Surgical complication data are extracted from Dekker et al. Efficacy data are extracted from Janssen. Patient counts are not additive between these two entries. ^‡^*N* = 280 represents the resection cohort analyzed for surgical complications (142 FOLFIRINOX, 138 gemcitabine CRT); total trial enrollment was 375. ^§^Total enrollment was 298; the comparative analysis included 152 patients (43 neoadjuvant CRT, 109 upfront surgery) after exclusions. BR, borderline resectable; BRPC, borderline resectable pancreatic cancer; BR-A, borderline resectable with arterial involvement; FU, follow-up; NR, not reported; PCG, pancreatic cancer group; Retro, retrospective

Study	Year	Country	Design	Center	N	BRPC details	BR definition	FU (Months)	Funding	Ref.
Janssen et al. (PREOPANC-2)	2025	The Netherlands	Phase-III RCT	Multi	375	Mixed (128 BRPC)	Dutch PCG	42.3	Dutch Cancer Soc, ZonMw	[4]
Pouypoudat et al.	2019	France	Retro cohort	Single	79	Mixed (41 BRPC)	NCCN v2-2017	NR	NR	[5]
Ghaneh et al. (ESPAC-5F)	2023	United Kingdom, Germany	Phase II RCT	Multi	90	Only BRPC	NCCN	NR	NR	[6]
Reni et al. (CASSANDRA)	2025	Italy	Phase III RCT	Multi	261	Mixed (134 BRPC)	NCCN + Biological	NR	MyEverest, Codice Viola	[7]
Thakur et al.	2025	India	Phase-II RCT	Single	20	Mixed (5 BRPC)	NR	NR	NR	[27]
Dekker et al. (PREOPANC-2)^†^	2024	The Netherlands	RCT sub-analysis	Multi	280^‡^	Mixed (86 BRPC)	Dutch PCG	NR	Dutch Cancer Soc, ZonMw	[28]
Okada et al.	2020	Japan	Retro cohort	Single	118	Only BRPC	NCCN	NR	NR	[29]
Parsonson et al.	2021	Australia	Retro cohort	Multi	48	Only BRPC	Variable	15.0	NR	[30]
Huang et al.	2026	China	Retro cohort	Single	298^§^	Only BRPC	NR	NR	None	[31]
Akahori et al.	2024	Japan	Retro cohort	Single	81	Only BRPC (BR-A)	NR	22.5	None	[32]
Barrak et al.	2022	United States	Retro database	Multi	5402	Mixed (NR)	N/A	NR	None	[33]
Kim et al.	2020	United States	Retro cohort	Single	541	Mixed (315 BRPC)	NR	NR	NR	[34]
Botta et al.	2022	United States	Retro cohort	Single	52	Mixed (33 BRPC)	NCCN	NR	NIH NCATS	[35]
Weniger et al. (RESPECT)	2020	Europe	Retro cohort	Multi	239	Mixed (NR)	NCCN	20.5	German Research Assoc	[36]
Nerwal et al.	2025	Australia	Retro cohort	Single	37	Mixed (NR)	NCCN	NR	Institutional	[37]
Thalji et al.	2023	United States	Retro cohort	Single	326	Mixed (134 BRPC)	NR	NR	NR	[38]
Neibart et al.	2021	United States	Retro cohort	Single	31	Mixed (27 BRPC)	NR	NR	NIH NCATS	[39]
Yamada et al.	2024	Japan	Retro cohort	Multi	687	Mixed (NR)	NCCN v2 2022	NR	NR	[40]
Mellon et al.	2015	United States	Retro cohort	Single	159	Mixed (110 BRPC)	AHPBA/SSAT/SSO	14.0	NR	[41]

*TNT vs. Standard NACRT* *: OS*

Two studies provided HRs for the direct comparison of TNT vs. standard NACRT. Barrak et al. analyzed 2,652 patients from the National Cancer Database and reported a significant survival advantage for TNT over CRT (median OS 23.2 vs. 19.9 months; HR (95% CI): 0.82 (0.72-0.94), *P* = 0.0049) [33]. Kim et al. compared TNT (*n* = 89) to short-course neoadjuvant therapy (*n* = 452) and found no statistically significant difference (HR (95% CI): 0.90 (0.50-1.90), *P* = 0.87) [34]. The wide CI in the analysis by Kim et al. indicates limited statistical power in the TNT arm (pooled HR (95% CI): 0.82 (0.72-0.94), *I*² = 0%, *P* = 0.003) (Figure 2).

**Figure 2:**
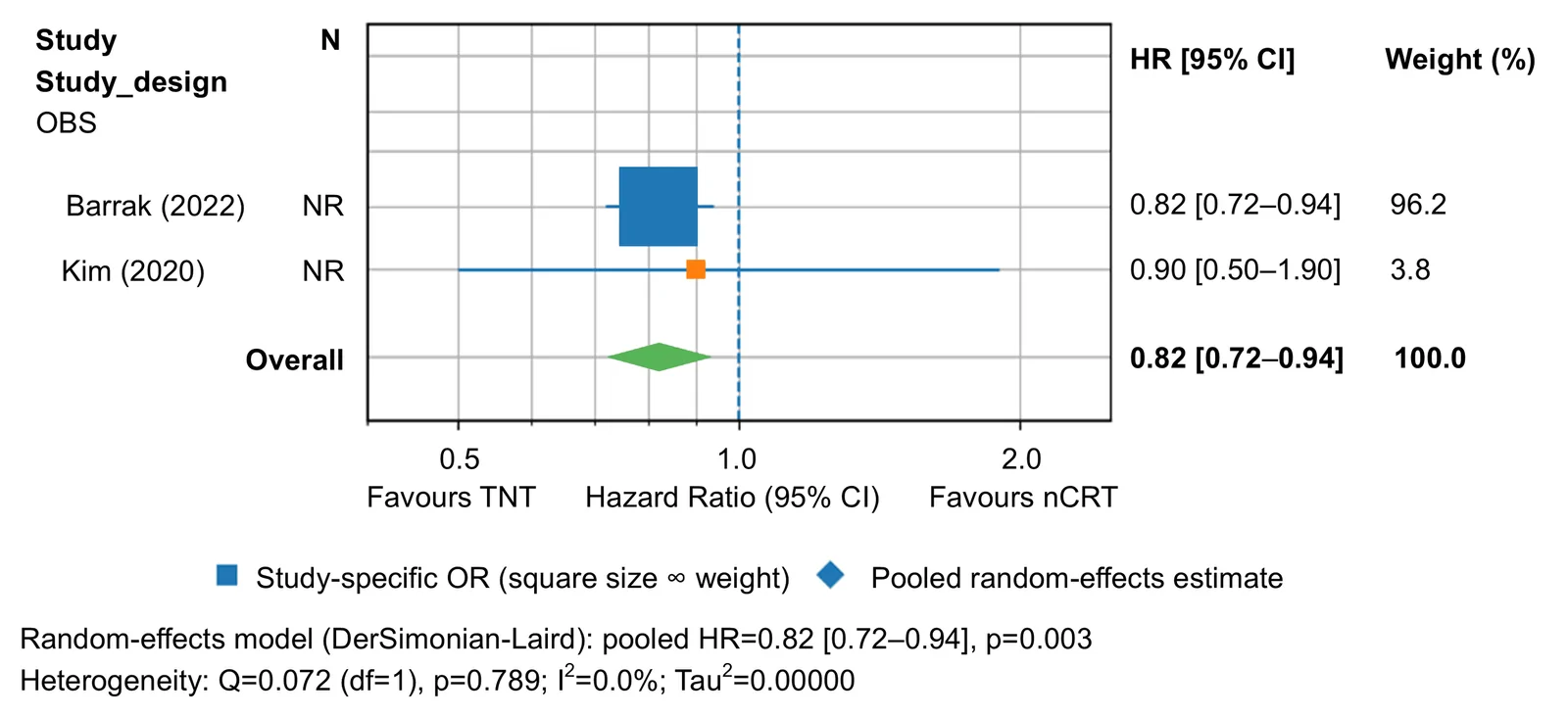
Forest plot of TNT vs. standard NACRT: OS. CI, confidence interval; df, degrees of freedom; HR, hazard ratio; NACRT, neoadjuvant chemoradiotherapy; NR, not reported; OBS, observational; OS, overall survival; TNT, total neoadjuvant therapy

In the BRPC subgroup of PREOPANC-2, Janssen et al. reported a numerically higher median OS for FOLFIRINOX compared with gemcitabine-based CRT (23.4 vs. 17.0 months; HR (95% CI): 0.80 (0.53-1.21), *P* = 0.30) [4,5]. The difference did not reach statistical significance. Akahori et al. demonstrated significantly better OS for TNT compared with standard NACRT by Kaplan-Meier analysis (*P* = 0.016) in 81 BRPC patients with arterial involvement, although an HR was not reported [32].

*TNT vs. Standard NACRT* *: R0 Resection Rate*

TNT was consistently associated with superior margin-negative resection rates. Kim et al. reported R0 rates of 94% (60/64 resected) for TNT vs. 80% (258/322) for SNT [34]. Akahori et al. observed an R0 rate of 95% (21/22 resected) for TNT compared with 78% (18/23) for NACRT [32]. Overall resection rates were similar or slightly lower for TNT, consistent with the concept that TNT serves as a period of biological observation, identifying patients with rapidly progressive disease who are unlikely to benefit from surgical intervention (pooled OR (95% CI): 4.03 (1.56-10.43), *I*² = 0%, *P* = 0.004) (Figure 3).

**Figure 3:**
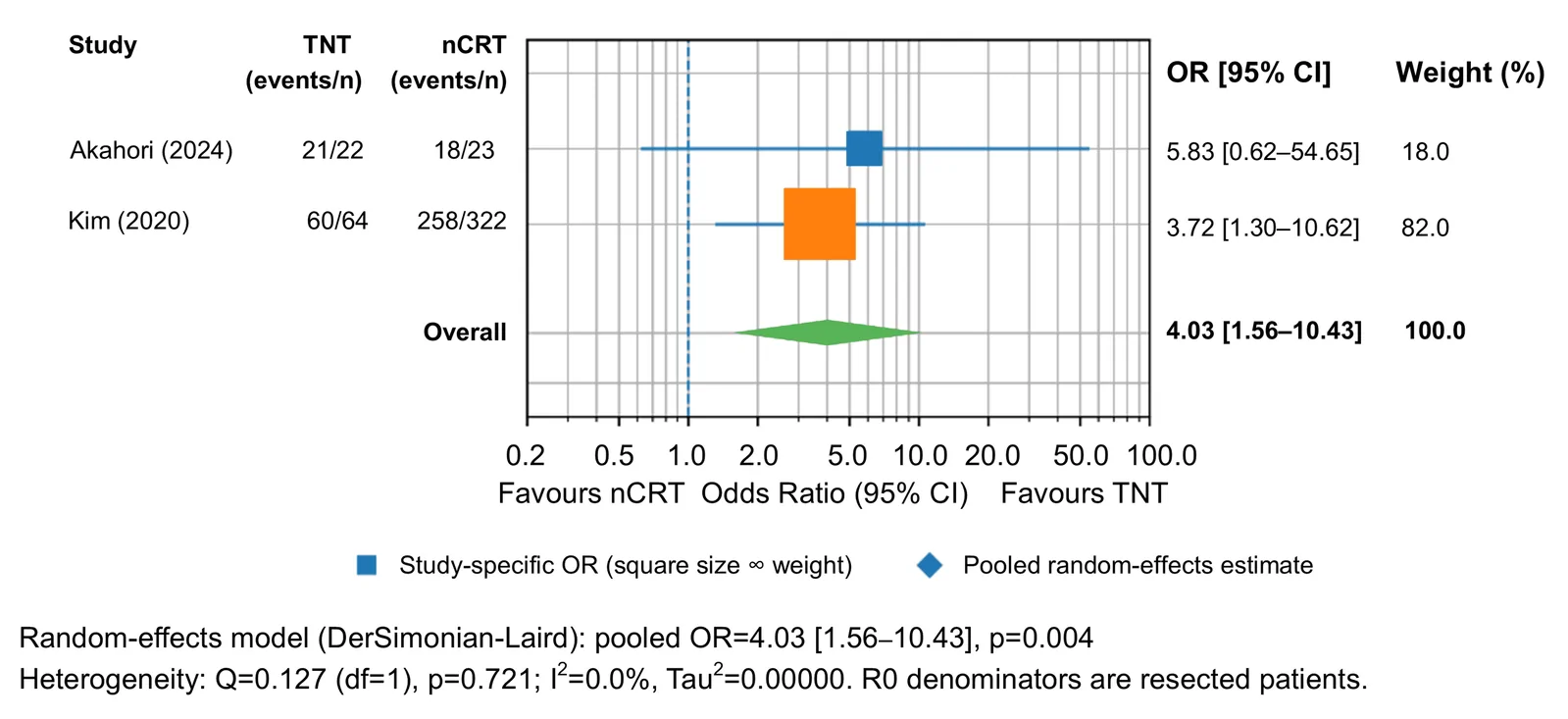
Forest plot of TNT vs. standard NACRT - R0 resection rate (OR). CI, confidence interval; df, degrees of freedom; NACRT, neoadjuvant chemoradiotherapy; OR, odds ratio; TNT, total neoadjuvant therapy

*TNT vs. Standard NACRT*​​​​​​​*: pCR*

pCR rates favored TNT across all reporting studies. Kim et al. found a pCR rate of 13% (8/64 resected) for TNT vs. 6% (20/322) for SNT [34]. Barrak et al. reported a pCR rate of 5.8% (79/1373) for TNT compared with 3.2% (41/1279) for CRT [33]. The Barrak denominator includes all enrolled patients (intention-to-treat); Kim et al. reported pCR among resected patients only [34]. This difference in denominator definition should be considered when interpreting the pooled estimate (pooled OR (95% CI): 1.89 (1.33-2.69), *I*² = 0%, *P* < 0.001) (Figure 4).

**Figure 4:**
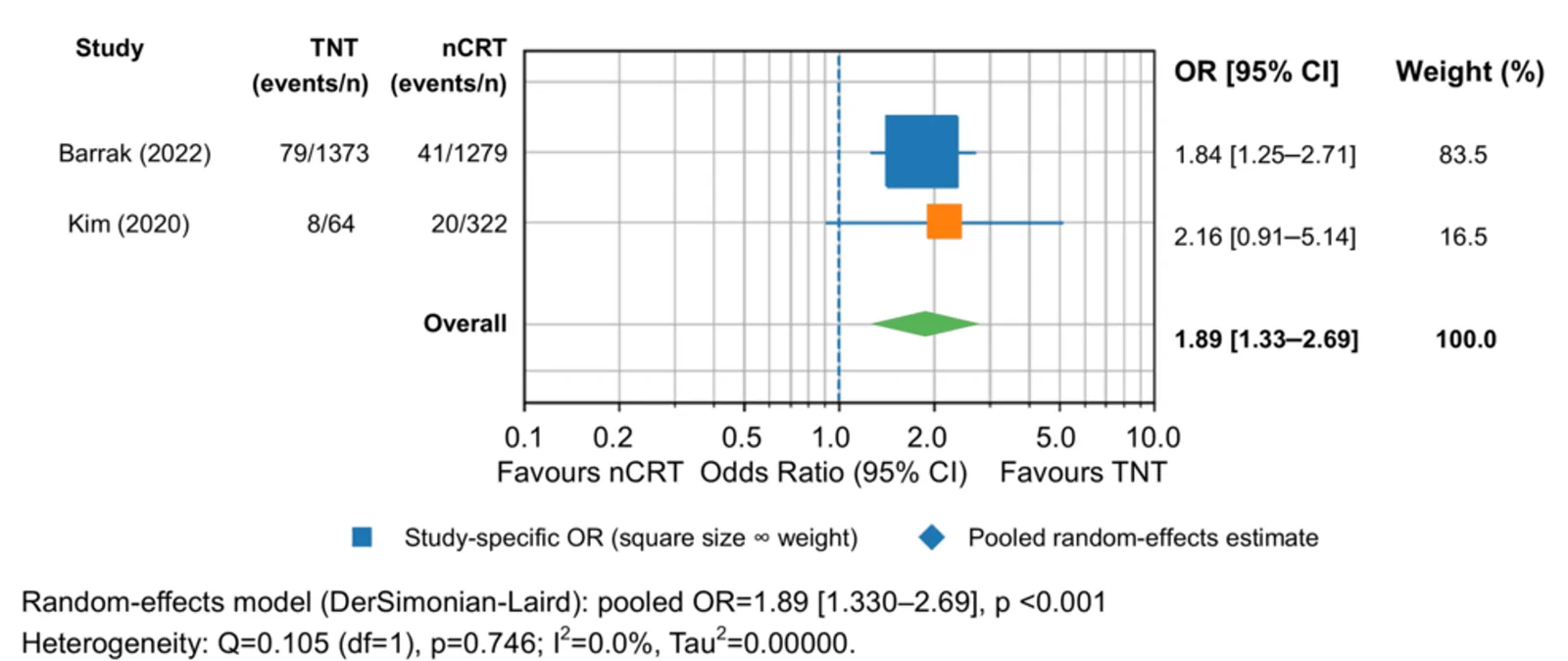
Forest plot of TNT vs. standard NACRT - pCR (OR). Barrak et al. [33] used the ITT denominator; Kim et al. [34] used the resected cohort denominator. CI, confidence interval; df, degrees of freedom; ITT, intention-to-treat; NACRT, neoadjuvant chemoradiotherapy; OR, odds ratio; pCR, pathologic complete response; TNT, total neoadjuvant therapy

*TNT vs. Standard NACRT*​​​​​​​*: Resection Rate*

Overall resection rates were statistically comparable. Kim et al. reported rates of 72% (64/89) for TNT and 71% (322/452) for SNT [34]. Akahori et al. found rates of 67% (22/33) for TNT and 77% (23/30) for NACRT [32]. The lower raw resection rate for TNT in Akahori et al. is consistent with the selection effect described above (pooled OR (95% CI): 0.94 (0.60-1.50), *I*² = 0%, *P* = 0.807) (Figure 5).

**Figure 5:**
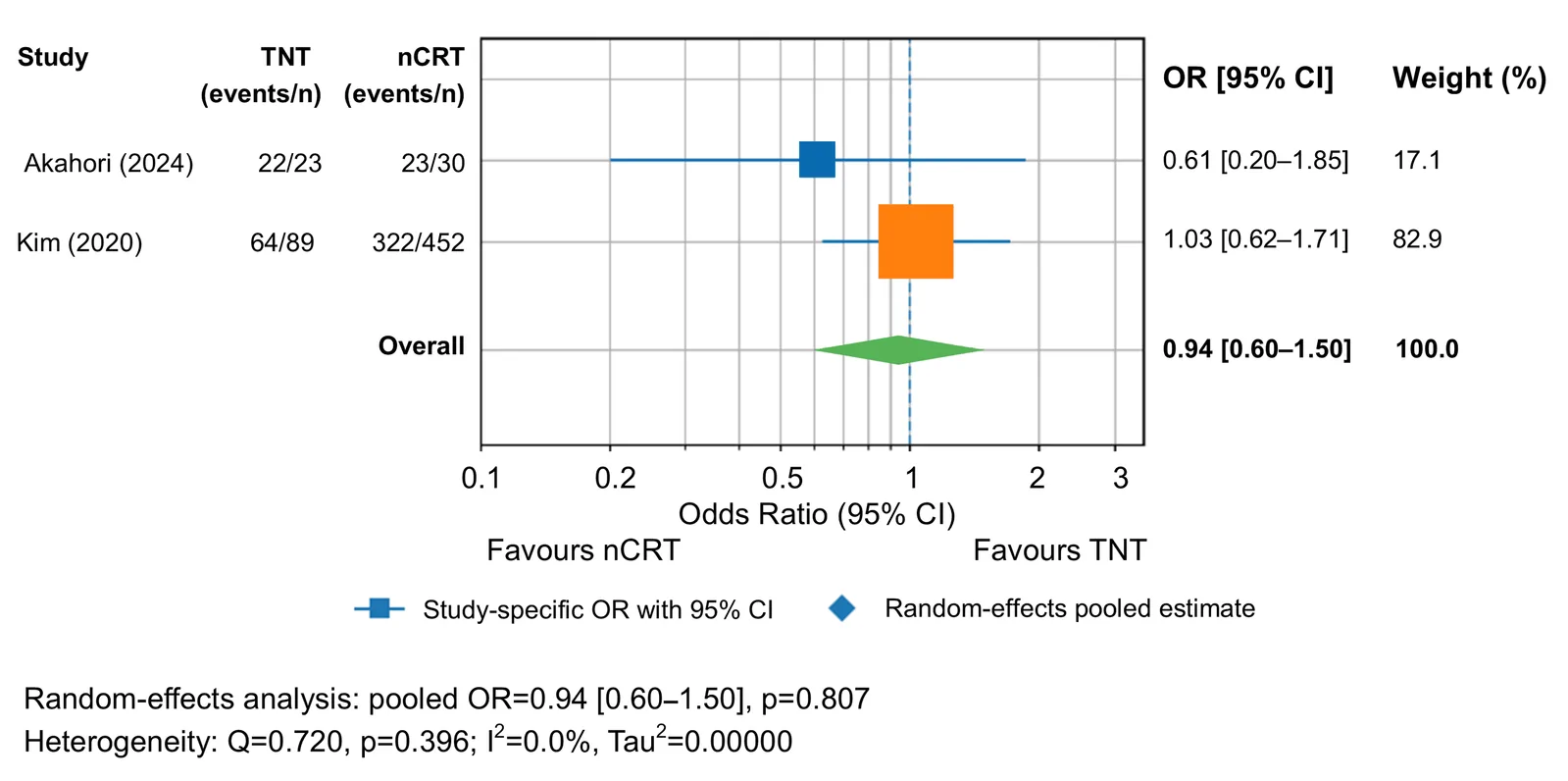
Forest plot of TNT vs. standard NACRT - resection rate (OR). CI, confidence interval; NACRT, neoadjuvant chemoradiotherapy; OR, odds ratio; TNT, total neoadjuvant therapy

Recurrence

Limited recurrence data were available. Kim et al. reported a locoregional recurrence rate of 0% for TNT vs. 2% for SNT [34]. This finding is consistent with the expected local cytoreductive effect of the radiotherapy component within the TNT sequence.

Does Radiotherapy Add Survival Benefit?

The PREOPANC-2 trial is the single randomized study that directly compares systemic chemotherapy alone (FOLFIRINOX, no RT) with concurrent CRT (gemcitabine-based CRT). In the BRPC subgroup, median OS was 23.4 months for FOLFIRINOX and 17.0 months for gemcitabine CRT (HR (95% CI): 0.80 (0.53-1.21), *P* = 0.30) (Figure 6) [4]. The direction of effect favored the chemotherapy-alone arm. This comparison is confounded by the different chemotherapy backbones in each arm; the observed difference may indicate the superiority of FOLFIRINOX as a systemic agent rather than the inferiority of radiotherapy [5].

**Figure 6:**
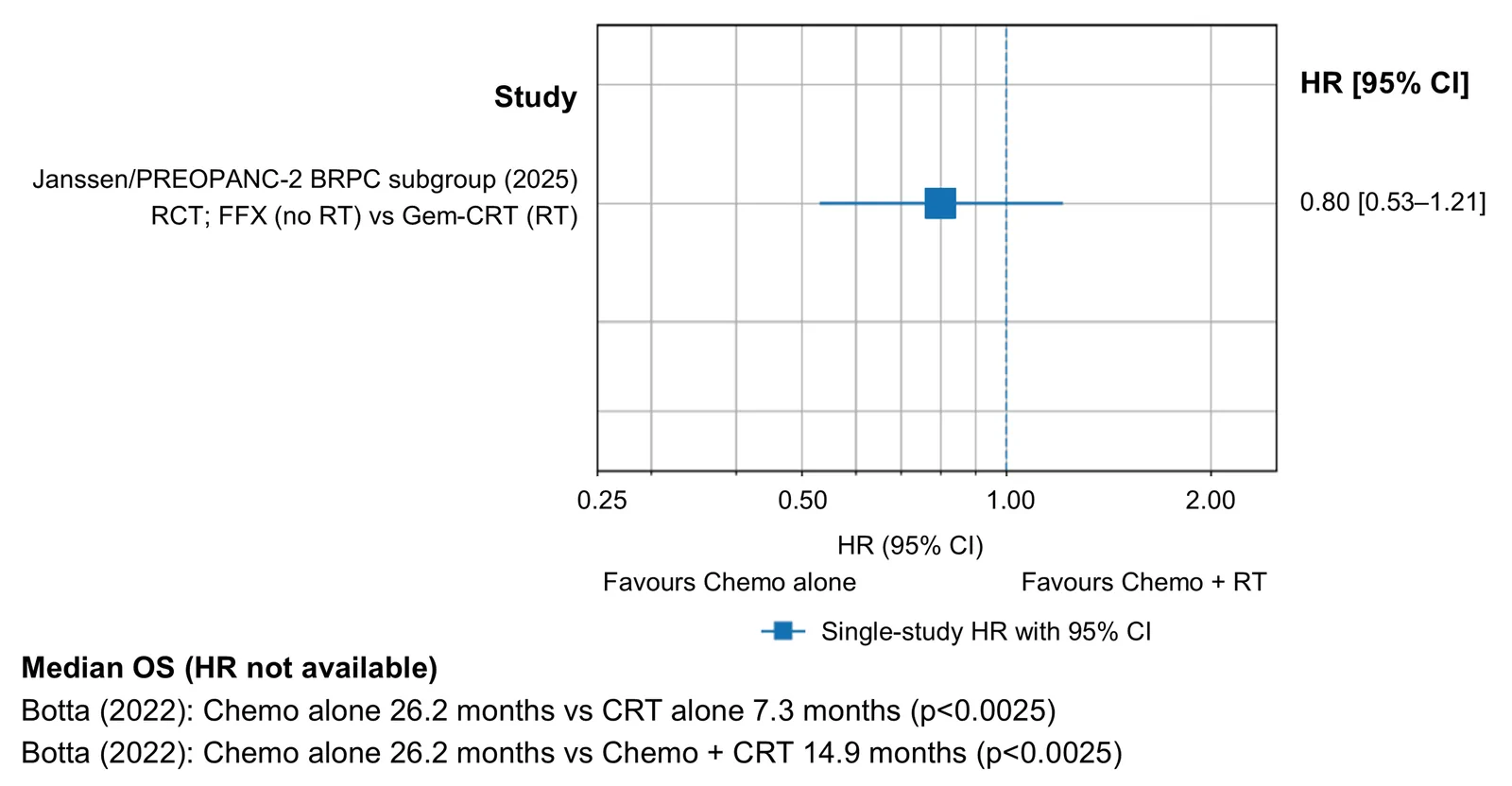
Forest plot of chemo alone vs. chemo + CRT - OS (HR). KM-derived; HR not reported. Formal pooling was not possible because only one HR estimate was available. BRPC, borderline resectable pancreatic cancer; CI, confidence interval; FFX, FOLFIRINOX; Gem, gemcitabine; HR, hazard ratio; CRT, chemoradiotherapy; OS, overall survival; RCT, randomized controlled trial; RT, radiotherapy; TNT, total neoadjuvant therapy

Botta et al. reported markedly higher median OS for chemotherapy alone (26.2 months) compared with sequential chemotherapy followed by CRT (14.9 months) and concurrent CRT alone (7.3 months) in a mixed BRPC/LAPC cohort (*P* < 0.0025) [35]. The resection rate was 50% in the chemotherapy arm but 8% in both radiation-containing arms. These findings are heavily confounded by selection bias.

The pattern across the evidence base indicates that radiotherapy improves local control and R0 rates but does not independently improve OS. Ghaneh et al. showed an R0 rate of 37% for the CRT arm compared with 18% for FOLFIRINOX among resected patients, despite a lower one-year OS for CRT (60% vs. 84%) (Figure 7) [6]. Wu et al. synthesized 31 prospective studies and found that neoadjuvant chemotherapy alone was associated with significantly longer OS than chemotherapy combined with CRT (25.6 vs. 17.6 months, *P* < 0.0001) [8]. CRT produced significantly higher R0 rates (83% vs. 70%). The survival disadvantage of CRT disappeared when five or more cycles of induction chemotherapy preceded radiation [8]. Saha et al. reviewed 34 publications and concluded that CRT after induction chemotherapy improves pathologic response and local control, although OS results were conflicting [24]. Turner et al. reviewed the radiation therapy evidence in BRPC specifically and found no clear survival benefit from RT when added to modern chemotherapy regimens [25].

**Figure 7:**
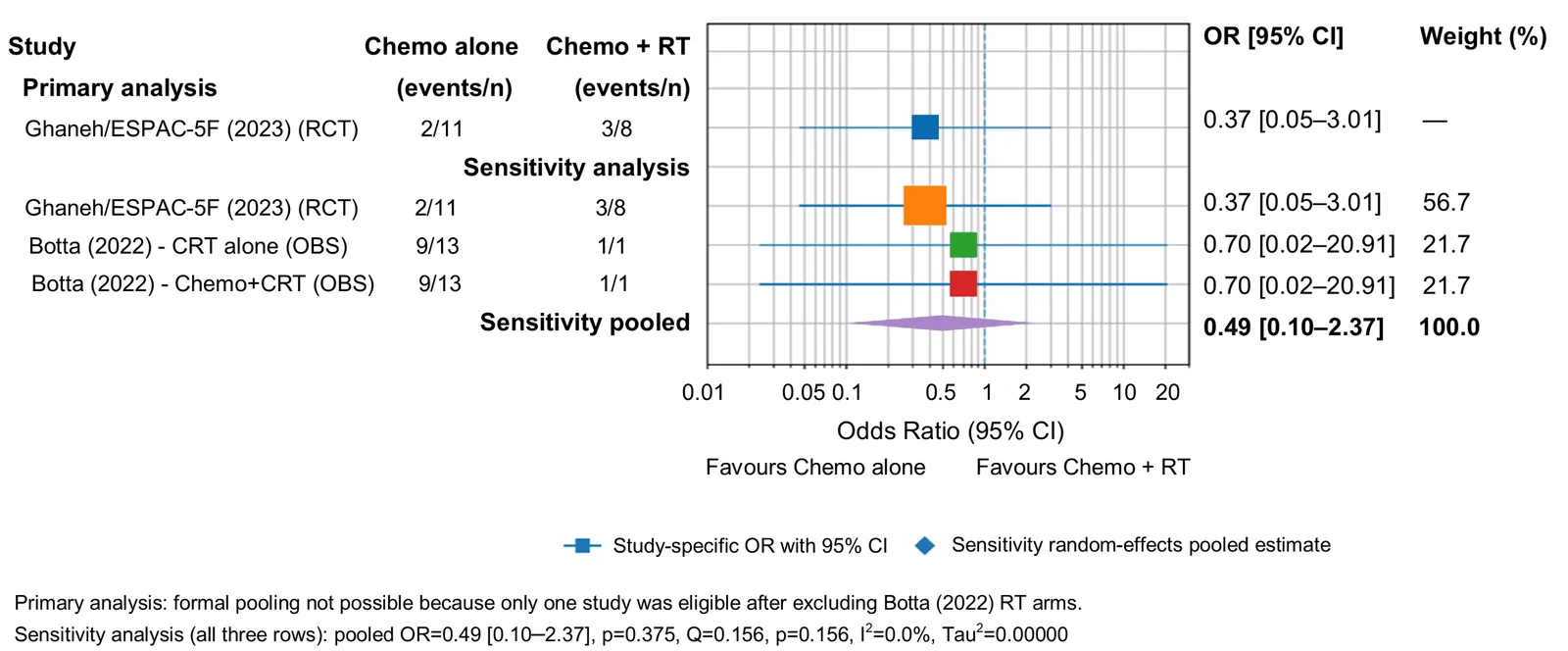
Forest plot of chemo alone vs. chemo + RT - R0 resection rate (OR). Botta et al. [35] arms were excluded from the primary pooling due to an *N* = 1 denominator in the RT arms. These rows are shown as sensitivity analysis only. ORs are unstable. The Haldane 0.5 correction was applied where needed. CI, confidence interval; CRT, chemoradiotherapy; OBS, observational study; OR, odds ratio; pCR, pathologic complete response; RCT, randomized controlled trial; RT, radiotherapy

These data suggest that the benefit of radiotherapy in BRPC is conditional on adequate prior systemic therapy. Radiation appears to maximize local tumor control and margin clearance. It does not replace the need for extended systemic chemotherapy to address distant micrometastatic disease.

Chemotherapy Backbone

The CASSANDRA trial directly compared mFOLFIRINOX and PAXG in resectable and borderline resectable PDAC. PAXG produced significantly longer median event-free survival (16.0 vs. 10.2 months; HR (95% CI): 0.63 (0.47-0.84), *P* = 0.0018) [7]. This is the only phase III trial to show a clear difference between two multi-agent neoadjuvant regimens in this population.

Weniger et al. retrospectively compared FOLFIRINOX, gemcitabine/nab-paclitaxel, and gemcitabine monotherapy in 239 patients across seven European centers [36]. Resection rates were 76% for FOLFIRINOX, 84% for GnP, and 44% for gemcitabine monotherapy. Median OS did not differ significantly between FOLFIRINOX and GnP (22.0 vs. 27.2 months, *P* = 0.23). R0 rates among resected patients were 52% for FOLFIRINOX and 44% for GnP (Figure 8).

**Figure 8:**
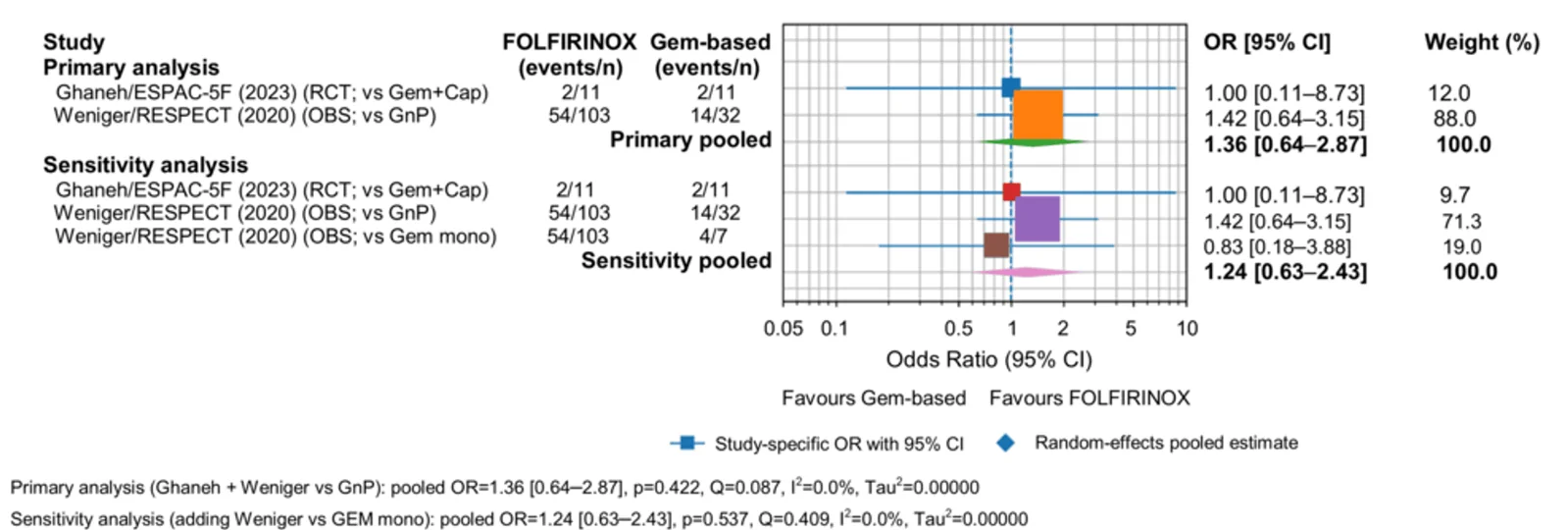
Forest plot of FOLFIRINOX vs. gemcitabine-based therapy - R0 resection rate. For formal primary pooling, only one comparator per study was used to avoid double-counting the Winger/RESPECT FOLFIRINOX arm. The FFX vs. GEM mono comparison is shown as a sensitivity analysis. CI, confidence interval; FFX, FOLFIRINOX; GnP, gemcitabine/nab-paclitaxel; OBS, observational study; OR, odds ratio; RCT, randomized controlled trial

In ESPAC-5F, Ghaneh et al. found comparable one-year OS between FOLFIRINOX (84%) and gemcitabine plus capecitabine (78%), with identical R0 rates of 18% among resected patients [6]. The low R0 rates across all ESPAC-5F arms likely indicate a stricter margin definition (1-mm clearance standard used in the UK) rather than treatment inefficacy.

Differential toxicity between backbones is relevant. Reni et al. reported grade 3-4 neutropenia in 36% of patients receiving PAXG vs. 24% of those receiving mFOLFIRINOX [7]. Weniger et al. observed grade 3-4 AEs in 10% of patients receiving FOLFIRINOX and 3% of those receiving GnP [36].

Efficacy outcomes of all compared studies are summarized in Table 2.

**Table 2:** Efficacy outcomes of comparative studies. mOS, median overall survival; HR, hazard ratio; CI, confidence interval; FFX, FOLFIRINOX; CRT, chemoradiotherapy; TNT, total neoadjuvant therapy; SNT, short neoadjuvant therapy; NACRT, neoadjuvant chemoradiotherapy; UFS, upfront surgery; GnP, gemcitabine/nab-paclitaxel; EFS, event-free survival; KM, Kaplan-Meier; NR, not reported

Study	Comparison	Arm 1 (*n*)	Arm 2 (*n*)	mOS Arm 1 (months)	mOS Arm 2 (months)	OS HR (95% CI)	R0 Arm 1	R0 Arm 2	pCR Arm 1 vs. Arm 2	Ref.
Janssen et al. (2025)	FFX vs. Gem-CRT	FFX (65)	CRT (63)	23.4	17.0	0.80 (0.53-1.21)	NR	NR	NR	[4]
Ghaneh et al. (2023)	FFX vs. CRT	FFX (20)	CRT (16)	NR	NR	NR	18%	37%	NR	[6]
Ghaneh et al. (2023)	FFX vs. GemCap	FFX (20)	GemCap (19)	NR	NR	NR	18%	18%	NR	[6]
Reni et al. (2025)	PAXG vs. mFFX	PAXG (132)	mFFX (128)	NR	NR	EFS: 0.63 (0.47-0.84)	NR	NR	NR	[7]
Huang et al. (2026)	CRT vs. UFS	CRT (43)	UFS (109)	33	17	0.56 (0.34-0.94)	70%	39%	4.7% vs. N/A	[31]
Akahori et al. (2024)	TNT vs. NACRT	TNT (33)	NACRT (30)	NR	NR	KM: *P* = 0.016	95%	78%	NR	[32]
Barrak et al. (2022)	TNT vs. CRT	TNT (1373)	CRT (1279)	23.2	19.9	0.82 (0.72-0.94)	NR	NR	5.8% vs. 3.2%	[33]
Barrak et al. (2022)	TNT vs. CT	TNT (1373)	CT (2750)	23.2	22.6	1.00 (0.88-1.14)	NR	NR	5.8% vs. 2.1%	[33]
Kim et al. (2021)	TNT vs. SNT	TNT (89)	SNT (452)	NR	25.1	0.90 (0.50-1.90)	94%	80%	13% vs. 6%	[34]
Botta et al. (2022)	Chemo vs. CRT	Chemo (26)	CRT (13)	26.2	7.3	KM: *P* < 0.003	69%	100% (1/1)	NR	[35]
Weniger et al. (2020)	FFX vs. GnP	FFX (135)	GnP (38)	22.0	27.2	KM: *P* = 0.23	52%	44%	NR	[36]
Thalji et al. (2023)	4 months vs. 2 months	4 months (88)	2 months (83)	31.1	24.8	0.78 (0.52-1.16)	91%	82%	9% vs. 4%	[38]

Safety and Surgical Morbidity

Grade 3-4 AEs during the neoadjuvant phase were common across all regimens (Table 3). Janssen et al. reported rates of 67% for FOLFIRINOX and 60% for gemcitabine-based CRT (CTCAE v4.03) [4]. Neutropenia was the most frequent severe toxicity in both arms (25% and 22%, respectively). Treatment-related mortality during the neoadjuvant phase was low at 1% in each arm.

**Table 3:** Treatment toxicity during neoadjuvant phase. AE, adverse event; Gr, grade; FFX, FOLFIRINOX; CRT, chemoradiotherapy; GnP, gemcitabine/nab-paclitaxel; NAT, neoadjuvant therapy; NR, not reported

Study	Arms	Gr 3-4 AE rate	Discontinuation	Completion rate	Top Gr 3-4 AEs	NAT mortality	Ref.
Janssen et al. (2025)	FFX vs. CRT	67% vs. 60%	NR	62% vs. 39%	Neutropenia 25%/22%, Diarrhoea 23%/NR	1% vs. 1%	[4]
Pouypoudat et al. (2019)	FFX then CRT	FFX 5%, CRT 3%	1.4%	CRT: 96%	Asthenia, diarrhoea	NR	[5]
Ghaneh et al. (2023)	Neoadjuvant vs. surgery	34% vs. 7%	NR	GemCap 100%, FFX 79%, CRT 86%	Neutropenia, infection	NR	[6]
Reni et al. (2025)	PAXG vs. mFFX	NR (aggregate)	NR	100% vs. 100%	Neutropenia 36%/24%	0% vs. 1%	[7]
Weniger et al. (2020)	FFX/GnP/GEM	10%/3%/38%	NR	NR	NR	NR	[36]
Nerwal et al. (2025)	NAT cohort	NR	5.4%	54%	Neutropenia 14%	NR	[37]
Mellon et al. (2015)	NAT cohort	7%	NR	NR	GI bleeding 4%	NR	[41]

Treatment completion rates varied. In PREOPANC-2, 62% of patients completed all planned FOLFIRINOX cycles vs. 39% for the CRT protocol [4]. Ghaneh et al. reported completion rates of 100% for gemcitabine plus capecitabine, 79% for FOLFIRINOX, and 86% for CRT [6]. Nerwal et al. observed that only 54% of patients in a mixed BRPC/LAPC cohort completed all planned neoadjuvant cycles [37].

The dedicated surgical complications analysis of PREOPANC-2 by Dekker et al. found comparable major morbidity between FOLFIRINOX (27%, 38/142) and gemcitabine-based CRT (28%, 38/138) [28]. FOLFIRINOX was associated with higher rates of grade B/C pancreatic fistula (10% vs. 4%) and post-pancreatectomy hemorrhage (8% vs. 4%). The CRT arm had higher in-hospital mortality (2.2% vs. 0%) and 90-day mortality (2.9% vs. 1.4%). Kim et al. reported lower major morbidity for TNT (3%, 2/62) compared with SNT (5%, 15/314) among resected patients (Table 4) [34].

**Table 4:** Surgical and postoperative morbidity. CD, Clavien-Dindo; POPF, postoperative pancreatic fistula; PPH, post-pancreatectomy hemorrhage; Mort, mortality; UFS, upfront surgery; NR, not reported; TNT, total neoadjuvant therapy; NACRT, neoadjuvant chemoradiotherapy; SNT, short neoadjuvant therapy; PAXG, cisplatin, nab-paclitaxel, capecitabine, and gemcitabine; mFFX, modified FOLFIRINOX

Study	Arms	Major morbidity (CD≥III)	POPF B/C	PPH	30d/In-hosp Mort	90d mortality	Ref.
Pouypoudat et al. (2019)	NAT cohort	14%	14%	5%	5%	NR	[5]
Ghaneh et al. (2023)	Neoadjuvant vs. Surgery	NR	NR	NR	0%	8% vs. 7%	[6]
Reni et al. (2025)	PAXG vs. mFFX	18% vs. 19%	NR	NR	1% vs. 0%	NR	[7]
Dekker et al. (2024)	FFX vs. CRT	27% vs. 28%	10% vs. 4%	8% vs. 4%	0% vs. 2.2%	1.4% vs. 2.9%	[28]
Huang et al. (2026)	CRT vs. UFS	40% vs. 32%	NR	NR	NR	NR	[31]
Akahori et al. (2024)	TNT/NACRT/UFS	18%/30%/19%	9%/22%/19%	NR	NR	NR	[32]
Kim et al. (2020)	TNT vs. SNT	3% vs. 5%	NR	NR	NR	NR	[34]
Weniger et al. (2020)	Overall	15%	8%	NR	0%	NR	[36]

Risk of Bias

Among the four RCTs assessed using Cochrane RoB 2 (Table 5, Figure 9), three received an overall judgment of *some concerns* [4,6,7]. All three studies were open-label; blinding was impractical in trials comparing different chemotherapy regimens or radiation modalities. Randomization was robust in all three, using centralized, computer-generated allocation with concealment. Attrition was minimal, and intention-to-treat analyses were performed. A trial by Thakur et al. received a judgment of *high risk *due to inadequate reporting of randomization methods, absence of blinded outcome assessment, and a sample size of only 20 patients [27].

**Table 5:** RoB assessment: randomized controlled trials (Cochrane RoB 2). D1-D5 correspond to the five RoB 2 domains. Low, low risk of bias; High, high risk of bias. RoB, risk of bias

Study	D1: Randomization	D2: Deviations	D3: Missing data	D4: Measurement	D5: Reporting	Overall	Ref.
Janssen et al. (2025) (PREOPANC-2)	Low	Some concern	Low	Low	Low	Some concerns	[4]
Ghaneh et al. (2023) (ESPAC-5F)	Low	Some concern	Low	Low	Low	Some concerns	[6]
Reni et al. (2025) (CASSANDRA)	Low	Some concern	Low	Low	Low	Some concerns	[7]
Thakur et al. (2025)	Some concerns	Some concerns	Low	Some concerns	Some concerns	High risk	[27]

**Figure 9:**
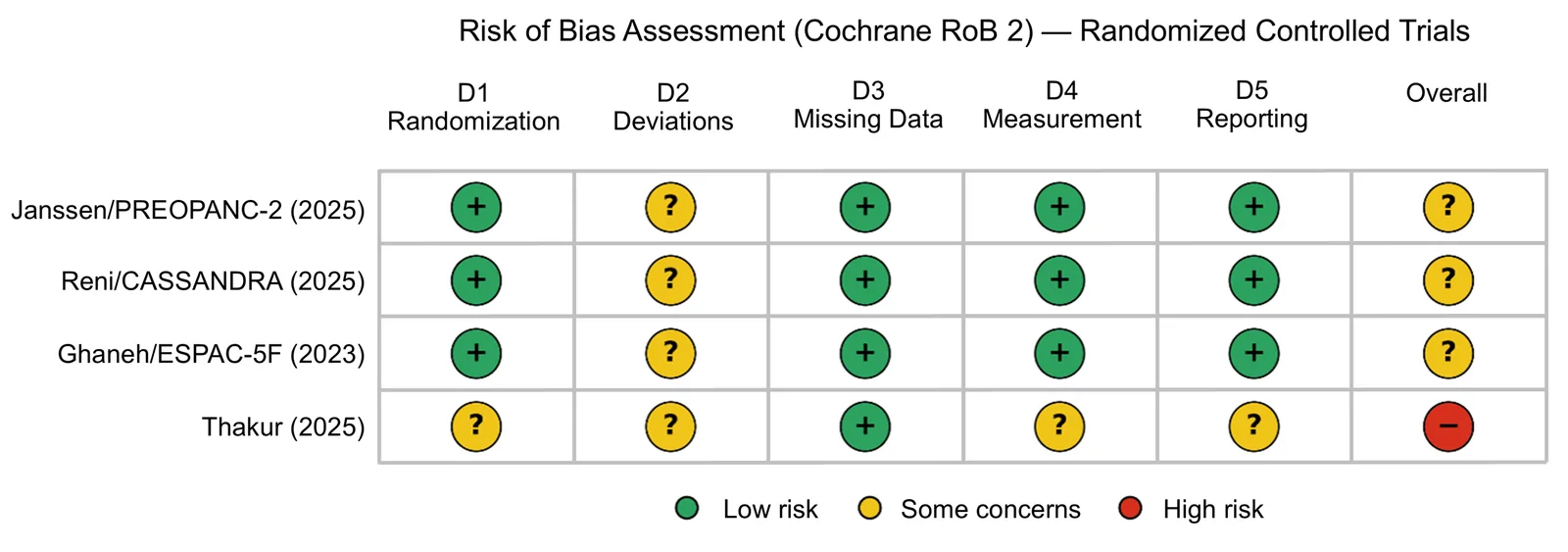
RoB traffic light plot (Cochrane RoB 2). RoB, risk of bias; HR, hazard ratio; NACRT, neoadjuvant chemoradiotherapy; OS, overall survival; TNT, total neoadjuvant therapy

Among the 14 observational studies assessed using the NOS (Table 6), six scored 7-9 (high quality) [31-34,38,39]. These studies performed multivariable Cox regression or propensity score adjustment. The remaining eight scored 4-6 (moderate quality), primarily losing points for comparability because of single-arm designs or the absence of confounder adjustment.

**Table 6:** Risk of bias assessment: observational studies (NOS). NOS scores: 0-3, low quality; 4-6, moderate quality; 7-9, high quality. NOS, Newcastle-Ottawa Scale

Study	Selection (out of 4)	Comparability (out of 2)	Outcome (out of 3)	Total (out of 9)	Quality	Key limitation	Ref.
Pouypoudat et al. (2019)	3	0	2	5	Moderate	Single-arm pilot study	[5]
Okada et al. (2020)	3	0	2	5	Moderate	Single arm; no comparator	[29]
Parsonson et al. (2021)	3	0	2	5	Moderate	Single arm; small sample size	[30]
Huang et al. (2026)	3	2	3	8	High	Single center	[31]
Akahori et al. (2024)	3	2	3	8	High	Small, single-center cohort	[32]
Barrak et al. (2022)	4	2	2	8	High	NCDB limits recurrence data	[33]
Kim et al. (2021)	3	2	3	8	High	Single center	[34]
Botta et al. (2022)	3	0	2	5	Moderate	No multivariable adjustment	[35]
Weniger et al. (2020)	4	0	2	6	Moderate	No multivariable adjustment	[36]
Nerwal et al. (2025)	3	0	2	5	Moderate	Single arm; no comparator	[37]
Thalji et al. (2023)	3	2	3	8	High	Single center	[38]
Neibart et al. (2021)	3	2	2	7	High	Single center; retro design	[39]
Yamada et al. (2024)	3	0	2	5	Moderate	No multivariate adjustment	[40]
Mellon et al. (2015)	3	0	2	5	Moderate	Single pathway; no comparator	[41]

Publication Bias

A funnel plot was generated for the primary OS analysis (Figure 10). With only two studies contributing HRs, formal assessment of publication bias using Egger's test was not feasible. Visual inspection was inconclusive because of the limited number of data points.

**Figure 10:**
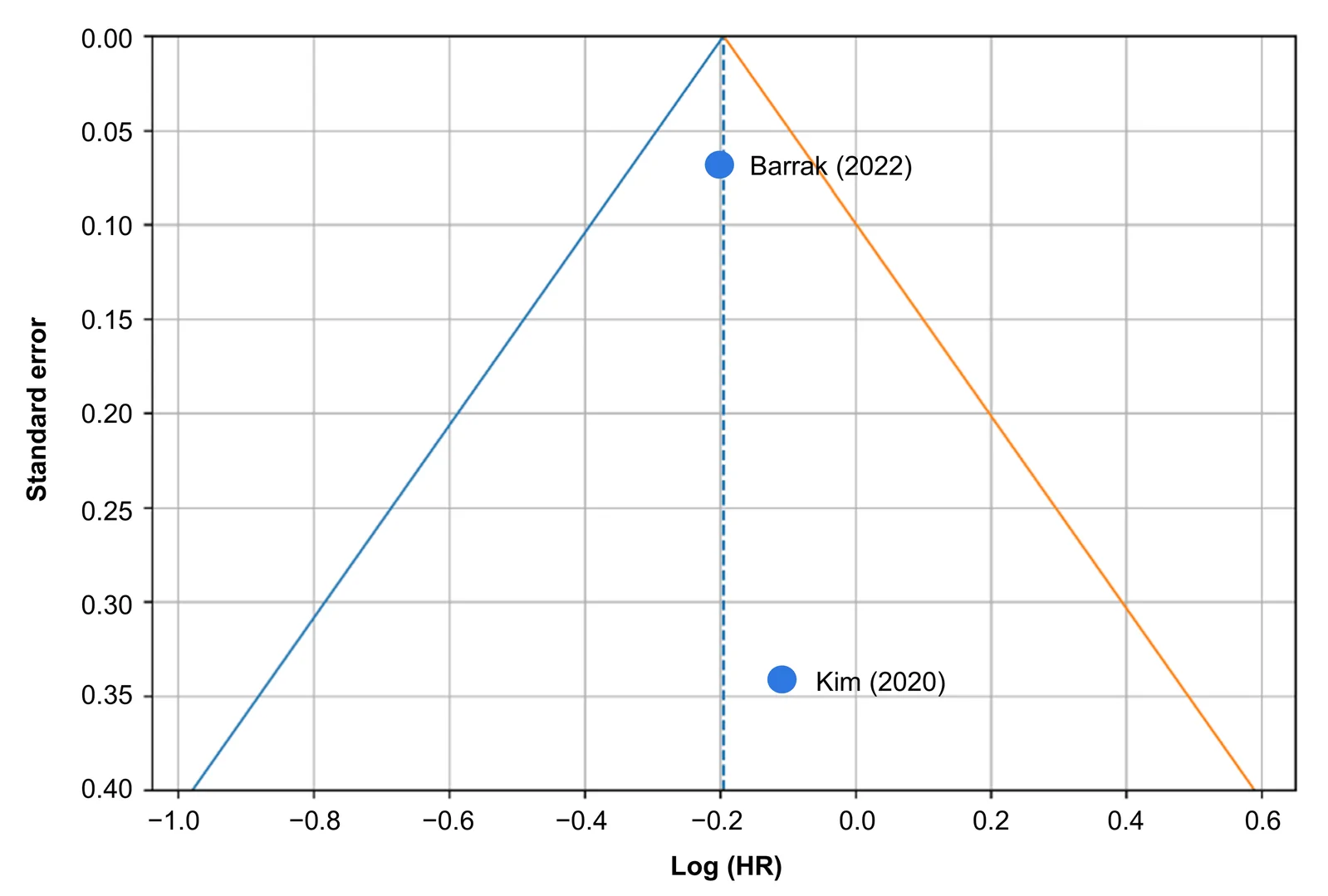
Funnel plot of TNT vs. NACRT - OS. NACRT, neoadjuvant chemoradiotherapy; OS, overall survival; TNT, total neoadjuvant therapy; HR, hazard ratio

Certainty of Evidence

The certainty of evidence for the two primary outcomes is summarized in Table 7. Both direct comparisons of TNT versus standard NACRT were informed exclusively by nonrandomized data, and both were rated as very low certainty. For OS, the pooled estimate favored TNT (HR: 0.82, 95% CI: 0.72-0.94; two studies, 3,193 patients; *I*^2^ = 0%). The evidence was downgraded for RoB and indirectness. For R0 resection rate, the pooled estimate strongly favored TNT (OR: 4.03, 95% CI: 1.56-10.43; two studies, 431 resected patients; *I*^2^ = 0%). This evidence was downgraded for RoB, indirectness, and imprecision. Margin status could be determined only in patients who proceeded to resection. The denominator was based on a post-treatment event and was not interchangeable between the arms; the definition of a negative margin also differed across the evidence base, with 1-mm clearance applied in some studies and tumor on ink in others. The CI was wide and was based on few events, with only 45 resected patients contributing data from Akahori et al. [32].

**Table 7:** GRADE summary of findings: TNT vs. standard NACRT in BRPC for the primary outcomes. ^a^Downgraded one level for risk of bias. Both contributing studies were nonrandomized. Although each was rated as high quality on the NOS and each adjusted for measured confounders, allocation to TNT was determined by clinician judgment and patient fitness. ^b^Not downgraded for inconsistency. *I*^2^ = 0% for both pooled analyses, and point estimates were directionally concordant. Note that with two studies per comparison, the power to detect heterogeneity is minimal. ^c^Downgraded one level for indirectness. Neither study reported a BRPC-restricted HR. ^d^Not downgraded for imprecision. The pooled CI excludes no effect and is derived from 3,193 patients with a large number of events. ^e^Not assessable. Fewer than 10 studies contributed to each analysis, so Egger's test was not feasible per protocol, and visual inspection of the funnel plot was inconclusive. ^f^Downgraded one level for risk of bias. In addition to the nonrandomized design and confounding described in footnote a, margin status was assessed by local pathologists without blinding to treatment allocation, and R0 was ascertained only among patients who underwent resection. ^g^Downgraded one level for indirectness. R0 resection is a surrogate for long-term survival. The denominator is restricted to resected patients, and the definition of a negative margin was not uniform across the evidence base (1-mm clearance vs. tumor on ink). ^h^Downgraded one level for imprecision. The pooled CI is wide (1.56-10.43) and spans effect sizes from modest to very large. Only 431 resected patients contributed, with sparse cell counts in Akahori et al. (21 of 22 vs. 18 of 23) [32], which inflates the odds ratio and widens the interval. CI, confidence interval; GRADE, Grading of Recommendations, Assessment, Development and Evaluations; HR, hazard ratio; NACRT, neoadjuvant chemoradiotherapy; OR, odds ratio; OS, overall survival; TNT, total neoadjuvant therapy

Outcome	No. of studies (participants)	Study design	Risk of bias	Inconsistency	Indirectness	Imprecision	Publication bias	Effect estimate (95% CI) and anticipated absolute effect	Certainty of evidence
OS	2 (3,193)	Observational studies	Serious^a^	Not serious^b^	Serious^c^	Not serious^d^	Not assessable^e^	HR 0.82 (0.72-0.94); *P* = 0.003; *I*^2^ = 0%; median OS 19.9 months with standard NACRT vs. 23.2 months with TNT (absolute difference 3.3 months longer)	Very low
R0 resection rate	2 (431 resected patients)	Observational studies	Serious^f^	Not serious^b^	Serious^g^	Serious^h^	Not assessable^e^	OR 4.03 (1.56-10.43); *P* = 0.004; *I*^2^ = 0%; 80 per 100 with standard NACRT vs. 94 per 100 with TNT (absolute difference 14 more per 100)	Very low

Definitions of certainty ratings: High: We are very confident that the true effect lies close to the estimate. Moderate: We are moderately confident; the true effect is likely to be close to the estimate but may be substantially different. Low: Our confidence is limited; the true effect may be substantially different. Very low: we have very little confidence; the true effect is likely to be substantially different from the estimate. Bodies of evidence consisting only of nonrandomized studies start at low certainty.

Neither outcome was downgraded for inconsistency: *I*^2^ was 0% for both pooled analyses, and the direction of effect was consistent across the contributing studies. However, with only two studies per comparison, heterogeneity statistics had very low power, and this domain should be interpreted with caution.

Discussion

This systematic review and meta-analysis synthesized evidence from 19 primary studies enrolling 9,219 patients. Three principal findings emerged from the analysis. TNT was associated with higher R0 resection and pCR rates compared with standard NACRT across both randomized and observational data. Kim et al. reported R0 rates of 94% vs. 80%, while Akahori et al. reported 95% vs. 78% [32,34]. The pCR rates consistently favored TNT (5.8%-13% vs. 2.1%-6.0%) [33,34]. These findings are biologically coherent. Extended systemic therapy provides greater tumor cytoreduction before surgery, while the addition of radiotherapy after induction chemotherapy targets residual local disease in a tumor already reduced in volume. The survival benefit was less consistent. Barrak et al. demonstrated a modest but significant advantage (HR, 0.82; *P* = 0.0049) [33], while Kim et al. found no significant difference (HR, 0.90; *P* = 0.87) [34]. This discrepancy may indicate survivorship bias; patients who tolerate several months of FOLFIRINOX followed by radiotherapy represent a favorably selected population.

The radiotherapy component did not demonstrate an independent survival benefit when added to systemic chemotherapy. The PREOPANC-2 BRPC subgroup analysis showed a nonsignificant trend favoring FOLFIRINOX alone over gemcitabine-based CRT (HR, 0.80; *P* = 0.30) [4]. This comparison is imperfect because it confounds chemotherapy backbone with radiation exposure. Botta et al. showed that chemotherapy alone produced superior OS to both sequential chemo-CRT and CRT alone, although selection bias limits causal inference [35]. Wu et al. found that chemotherapy alone produced significantly longer OS than combined chemotherapy and CRT (25.6 vs. 17.6 months) [8]. Radiation improved R0 rates (83% vs. 70%), and the survival disadvantage vanished when five or more cycles of induction chemotherapy preceded CRT [8]. This finding aligns with the biological logic: radiation controls local disease, but local control translates to a survival benefit only when systemic micrometastatic disease has already been addressed by adequate chemotherapy.

The choice of chemotherapy backbone affects event-free survival. The CASSANDRA trial is the first phase III trial to show superiority of one multi-agent regimen over another in this setting, with PAXG outperforming mFOLFIRINOX (HR, 0.63; *P* = 0.0018) [7]. Weniger et al. found comparable outcomes between FOLFIRINOX and GnP among resected patients [36]. The higher neutropenia rate with PAXG (36% vs. 24%) indicates a toxicity trade-off against the efficacy gain [7].

Strengths and Limitations

This review benefits from a comprehensive search across five databases, a preregistered protocol, and systematic RoB assessment. The inclusion of observational data alongside RCTs increases statistical power and captures real-world practice patterns but introduces selection bias and heterogeneity.

The number of studies contributing to each meta-analysis is small, particularly for the primary OS comparison (two studies with direct HRs). Several studies enrolled mixed populations, and BRPC-specific subgroup data were not always separately available. Definitions of borderline resectability varied across studies. The R0 margin definition also differed: 1-mm clearance in UK studies vs. tumor on ink in U.S. and European studies. Median follow-up was reported in fewer than one-third of studies. Toxicity reporting was inconsistent; many studies did not provide aggregate grade 3-4 AE rates.

The confounding of chemotherapy backbone with radiotherapy exposure in PREOPANC-2 limits causal inference about the independent role of radiation [4]. The Barrak et al. NCDB study, which contributes the largest patient volume and the most precisely estimated HR to the primary analysis, carries inherent limitations in data granularity, coding accuracy, and the inability to capture recurrence data [33].

Implications for Practice

These findings support a neoadjuvant approach that prioritizes extended multi-agent systemic chemotherapy as the backbone of treatment for BRPC. The evidence does not support routine addition of radiotherapy for all patients. Radiation may be most valuable when preceded by at least five cycles of induction chemotherapy and when the goal is local tumor control in patients who have demonstrated disease stability on systemic therapy. Selection of chemotherapy backbone should consider the emerging evidence favoring four-drug regimens such as PAXG, balanced against individual patient tolerance.

Implications for Research

Future randomized trials should directly compare TNT (extended chemotherapy followed by radiotherapy) against extended chemotherapy alone in BRPC, using a common chemotherapy backbone in both arms to isolate the independent contribution of radiation. The BRPCNCC-1 trial protocol provides a model for this design [26]. Standardization of borderline resectability definitions and R0 margin criteria across international trials would substantially reduce heterogeneity in future meta-analyses.

## Conclusions

TNT improves margin-negative resection rates and pCR rates compared with standard NACRT in BRPC. The OS benefit is modest and appears driven primarily by the extended systemic chemotherapy component rather than radiotherapy. Radiotherapy does not independently improve survival when added to systemic chemotherapy, although it enhances local control and R0 resection rates when preceded by adequate induction chemotherapy. The PAXG four-drug regimen demonstrated superior event-free survival over mFOLFIRINOX. Treatment decisions in BRPC should prioritize adequate duration of multi-agent systemic therapy; the role of radiotherapy should be individualized.
